# Supplementary material for: Decorated bacteria-cellulose ultrasonic metasurface
Source: Nat Commun. 2023 Sep 1;14:5319. doi: 10.1038/s41467-023-41172-2 (PMC10474036; doi:10.1038/s41467-023-41172-2)
Supplement: Supplementary file 1 — Supplementary Information [file 41467_2023_41172_MOESM1_ESM.pdf]

Supplementary Materials for

Decorated bacteria-cellulose ultrasonic metasurface

Zong-Lin Li[1,2*], Kun Chen[3*], Fei Li[2*], Zhi-Jun Shi[3], Qi-Li Sun[1], Peng-Qi Li[2], Yu-Gui Peng[1], Lai-Xin Huang[2], Guang Yang[3†], Hairong Zheng[2†], and Xue-Feng Zhu[1†]

[1]School of Physics and Innovation Institute, Huazhong University of Science and Technology, Wuhan, 430074, China.

[2]Shenzhen Institutes of Advanced Technology, and Biomedical Imaging Science and System Key Laboratory, Chinese Academy of Sciences, Shenzhen, 518055, China.

[3]College of Life Science and Technology, Huazhong University of Science and Technology, Wuhan, 430074, China.

[*]Zong-Lin Li, Kun Chen, and Fei Li contributed equally to this work.

[†]To whom correspondence should be addressed. Emails: yang_sunny@yahoo.com (G.Y.); hr.zheng@siat.ac.cn (H.R.Z.); xfzhu@hust.edu.cn (X.F.Z.).

Table of contents

Supplementary Note 1. Property and stability of BC meta-skin.........3
Supplementary Note 2. Bioactive property of BC meta-skin.........5
I. Materials.........5
II. Self-repairing experiment.........5
III. Self-degradation experiment.........8
Supplementary Note 3. Theoretical and simulation of BC meta-skin.........9
I. Model of superhydrophobic surfaces.........9
II. Simulation of total ultrasound reflection.........10
Supplementary Note 4. Theory and experiment of acoustic holography.........11
I. Theory.........11
II. Experiment.........14
Supplementary Note 5. Design principle of 3D ultrasound imaging.........16
I. Theory.........16
II. Experiment.........18
Supplementary Note 6. Lateral and longitudinal resolutions in imaging.........20
Supplementary Note 7. Technical recipes for ultrasound imaging.........22
I. Theory.........22
II. Experiment.........23
Supplementary References.........28

Supplementary Note 1. Property and stability of BC meta-skin

In this section, we show the superhydrophobicity of BC meta-skin by using a high-speed camera to record the process that a water droplet (diameter: 3 mm) impinged on the BC meta-skin surface, falling from a height of 5 cm and bouncing, as shown in Supplementary Fig. 1(a). Specifically, when a water droplet struck the fabricated BC film, we observed that the droplet did not stick to the surface, but remained with an intact volume in the bouncing process, clearly showing the superhydrophobicity (or the "lotus effect") of the decorated BC meta-skin. The process in Supplementary Fig. 1(a) is presented in the Supplementary Movie 1. We also presented Supplementary Movie 2 to prove the existence of tremendous surface energy of the BC meta-skin, in which a patch of meta-skin forced to immerse in water can jump high out of the liquid when the force was removed.

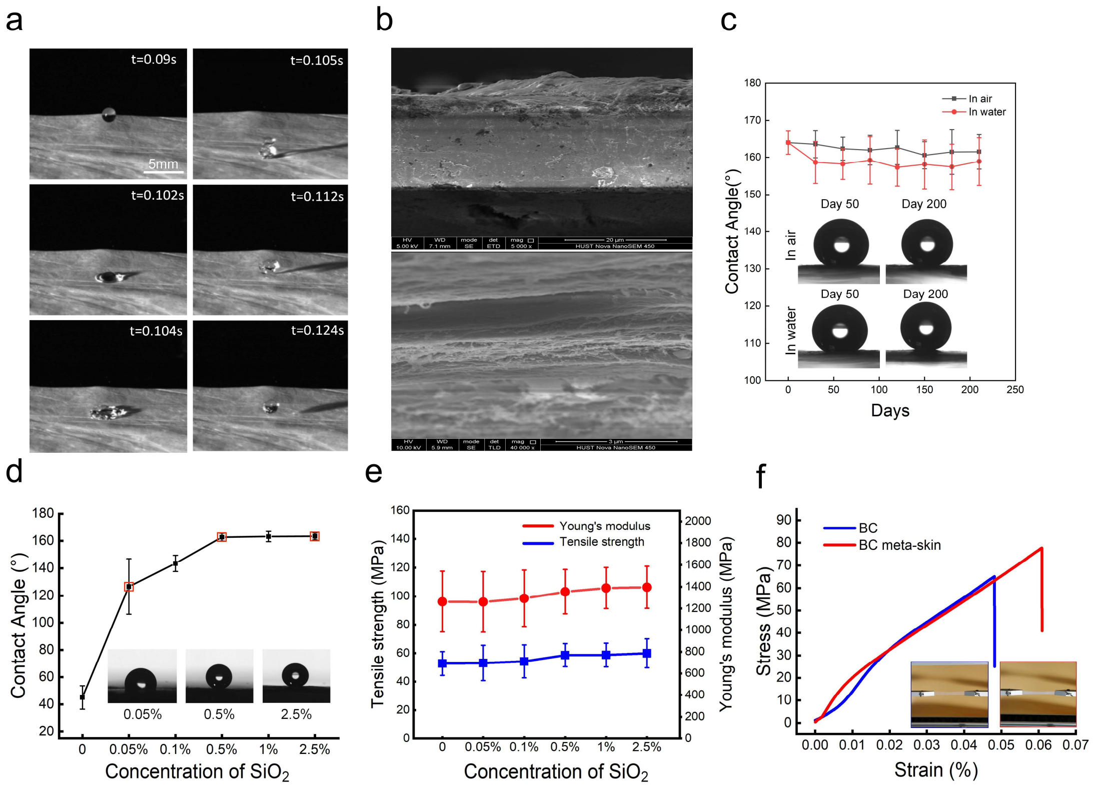

Supplementary Fig. 1. Superhydrophobicity and mechanical property of BC meta-skin. a, A water droplet impinging on the meta-skin, which was taken by a high-speed camera, indicating the existence of superhydrophobicity. **b,** Cross-sectional SEM

images of the BC meta-skin. **c**, The contact angle (CA) of a water droplet resting on the meta-skin surface. Within 200 days, the measurement of CA verified the stability of superhydrophobic property of BC meta-skin with/without immersing in water. **d**, The CA of decorated BC meta-skins prepared with different SiO_2 concentrations. **e**, Young's modulus and tensile strength of decorated BC meta-skins with different SiO_2 concentrations. **f**, The strain-stress curves of pure BC membrane and decorated BC meta-skin.

Figure S1(b) shows the SEM (scanning electron microscope) images of the BC meta-skin after being immersed in water for 200 days, indicating the swelling effect is nearly negligible since the measured thickness is still about 20 μm. In Supplementary Fig. 1(c), we display the performance life time of BC meta-skin for practical usages. In the life-time tests, we chose two groups, for which one was exposed in air and the other was immersed in water. The contact angles (CA) in the two cases were measured in a 25-day period within 200 days. The results in Supplementary Fig. 1(c) show that the superhydrophobic property of BC meta-skin is very robust. For example, in the air-matrix group, we have CA (50 days) = 162.3° and CA (200 days) = 161.5°, while in the water-matrix group we have CA (50 days) = 158.2° and CA (200 days) = 158.9°. We find that the superhydrophobic property of BC meta-skin is very stable in air, since the CA maintained over 160° for 200 days. In the water-matrix case, the property was slightly degraded after 25 days, but the CA still kept to be over 150°. The results reveal that the functional ultrasonic devices made by BC meta-skin paper-cutting have a very long life-time performance due to the stable micro/nano structure of SiO_2-pariticle-decorated BC network in air or water environments.

In Figs. S1(d)-(f), we further study the relation between the material properties and the SiO_2 concentrations in preparation. In the experiments, the samples were prepared with dispersion solution under different SiO_2-nanoparticle concentrations of 0% (Pure BC membrane), 0.05%, 0.1%, 0.5%, 1% and 2.5% (BC meta-skin). The measured average CAs of the samples are shown in Supplementary Fig. 1(d). The result shows

that with the concentration of SiO_2 nanoparticles increasing from 0% to 2.5%, the CAs of samples change from 45.0° to 163.2°. It is notable that the CA becomes stable for the samples with SiO_2 concentration above 0.5%, which is at around 160° and clearly indicates the existence of superhydrophobicity. In this work, the 2.5% SiO_2-nanoparticle dispersing solution was utilized to fabricate the decorated BC meta-skin.

In Supplementary Fig. 1(e), the measured results demonstrate that the mechanical properties of BC meta-skin are also enhanced by the decorative SiO_2 nanoparticles. To be specific, the Young's modulus and tensile strength of decorated BC meta-skins become larger as SiO_2 concentration increases, for which the average tensile strengths of pure BC membrane (0% SiO_2 concentration) and BC meta-skin (2.5% SiO_2 concentration) are 52.8 MPa and 59.9 MPa, respectively. In the tensile failure test, as shown in Supplementary Fig. 1(f), the strain-stress curves of BC membrane and meta-skin show that the maximum stresses undertook by the same-size samples are 65.1 MPa and 77.7 MPa, respectively.

Supplementary Note 2. Bioactive property of BC meta-skin

I. *Materials*

For the self-repairing experiment, we purchased the glucose, yeast extract, peptone, disodium hydrogen phosphate dodecahydrate, and citric acid monohydrate from Sigma Aldrich (Milwaukee, WI, USA). We also purchased the gluconacetobacter xylinum (G. xylinum, ATCC 53582, Gram-negative bacterium) from the American Type Culture Collection, ATCC.

The BC fibers in the degradation experiment were purchased from the Hainan Yide Foods Co. Ltd. (China). The cellulase was from Beijing Soleibao Technology Co., Ltd. Sodium acetate and acetic acid were bought from Shanghai Biochemical Technology Co., Ltd. All the samples of hydrophilic BC films and superhydrophobic BC meta-skins were prepared with the same size of 3×3 cm^2.

II. *Self-repairing experiment*

A piece of 3×3 cm^2 BC meta-skin was cut with holes to create the breakages for the self-repairing experiment (Supplementary Figs. 2(a), (d)). The damaged meta-skin was immersed in the Hestrin-Schramm (HS) medium inside a culture vessel, which was sealed by a filter film. Here the HS medium comprised 20 g/L glucose, 5 g/L yeast extract, 5 g/L peptone, 6.8 g/L disodium hydrogen phosphate dodecahydrate, and 1.5 g/L citric acid monohydrate. The sealed culture vessel was then placed in the clean bench and was sterilized by high temperature, high pressure and ultraviolet radiation. The G. xylinus seed was inoculated into the HS medium in the culture vessel at a volume ratio of 1:10. Finally, the culture vessel was placed in an incubator undisturbedly, while the damaged meta-skin finished the self-repairing process. After the above self-repairing process, the sample was taken out of the medium and put into 1 mol/L NaOH solution and ultrapure water in sequence to remove the residual G. xylinus and other impurities. After the post-process in preparation of BC meta-skins as shown in Fig. 1 of the main text, the self-repairing of BC meta-skins was finally finished (Supplementary Figs. 2 (b), (e)).

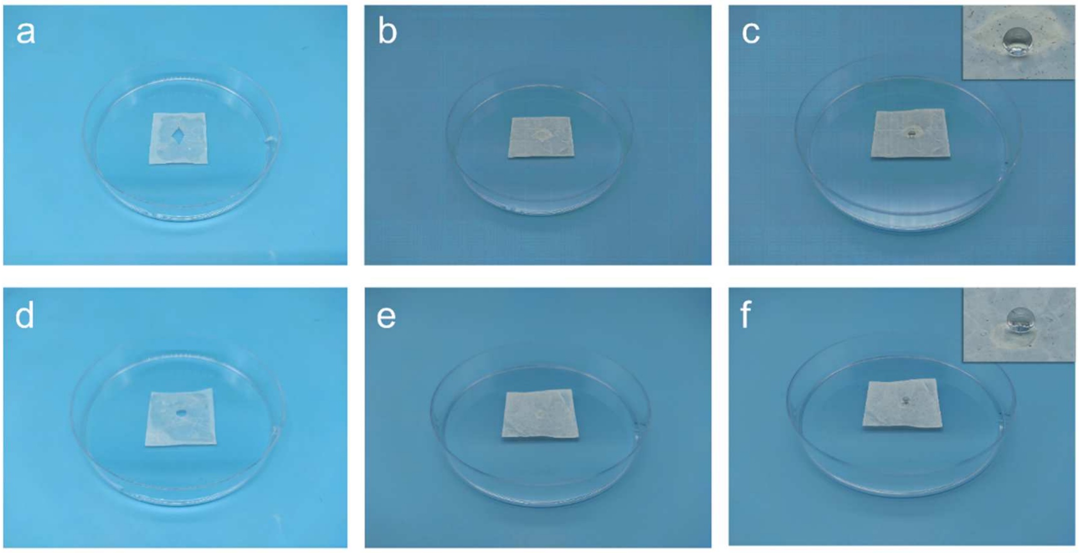

Supplementary Fig. 2. Self-repairing of BC meta-skin with different breakages. a, The broken sample with a diamond-shaped hole. **b**, The healed sample after the self-repairing process. **c**, The “lotus effect” on the surface of a healed sample. **d**, The broken sample with a circular hole. **e**, The healed sample after the self-repairing process. **f**, The “lotus effect” on the surface of a healed sample.

In this study, the basic raw material of BC membrane is secreted by bacteria (G. xylimum). Therefore, the self-repairing process also need the bacteria (G. xylimum) to act as a dressmaker to repair the damaged BC meta-skin. The bacteria (G. xylimum) play an important role in this search. It is the secretory behavior of bacteria that endows BC meta-skin with the excellent mechanical properties, degradability, fibrous network structure, and so on. In the self-repairing process, bacteria in the damaged part secrete newborn bacterial cellulose to repair the breakages.

The BC meta-skin can be regarded as a type of bioactive materials, for which the G. xylinus acts as dressmakers with the secretion behavior to repair the damaged BC meta-skins. The repaired BC meta-skin has the same superhydrophobic property as the perfect one, which supports the “lotus effect” on the nanosurface, as shown in Supplementary Figs. 2(c), 2(f). In Supplementary Movie 3, we observe that the surface of repaired BC meta-skin was silvery when immersed into water and became dry when taken out of water. As shown in the main text, the BC meta-skin possesses an extremely thin thickness (~20 μm), leading to its ultralight weight. For example, two different ultrasonic meta-lenses made by the meta-skin paper-cutting take the weights of 10.3 mg (3D-imaging meta-lens) and 18.6 mg (holographic meta-lens), as shown in Supplementary Fig. 3. In Supplementary Movie 4, we clearly show that the fabricated meta-lenses made of this approach can rest on the dandelion’s crest.

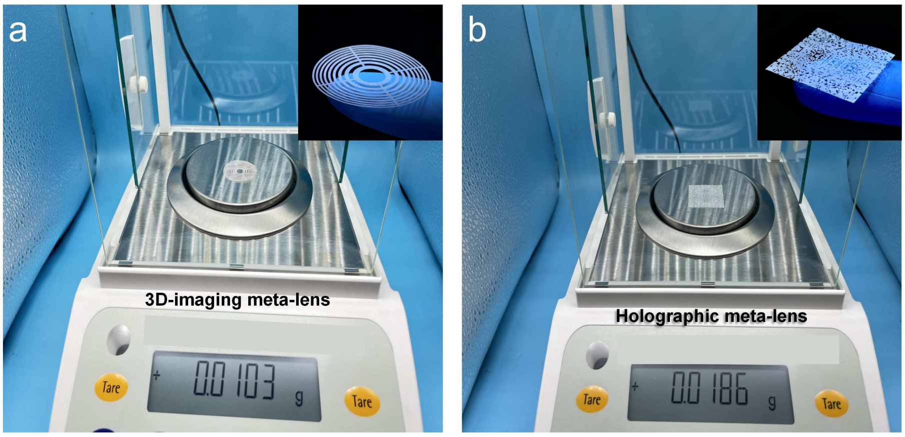

Supplementary Fig. 3. Measurement of meta-lens weights via a precision balance. **a**, The weight of the 3D-imaging meta-lens (10.3 mg). **b**, The weight of the holographic meta-lens (18.6 mg).

III. *Self-degradation experiment*

We prepared a HAc-NaAc buffer with a pH of 4.8 by mixing 150 mL of 0.2 mol/L sodium acetate mother liquor with 100 mL of 0.2 mol/L acetate mother liquor and then diluted the volume to 1 L with ultrapure water. 1 g cellulase was dissolved into 100 mL of HAc-NaAc buffer to obtain the enzyme solution. The hydrophilic BC films and superhydrophobic BC meta-skins were submerged into the enzyme solution (50 °C in a water bath) for degradation contrast experiments. The samples were photographed per hour to record the degradation process until the samples were completely degraded.

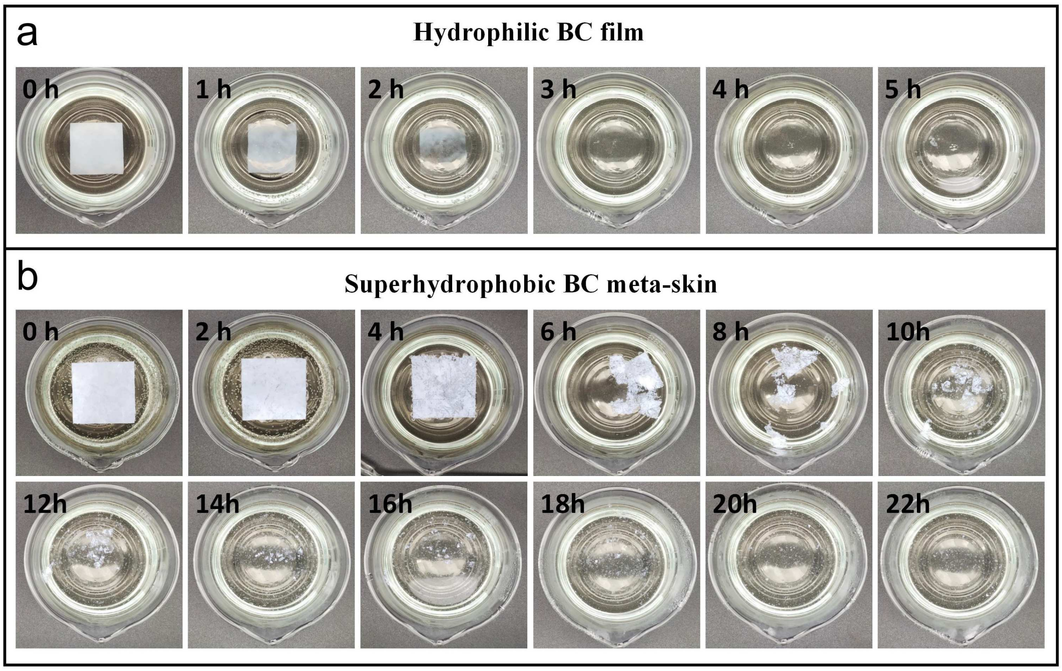

Supplementary Fig. 4. The bio-degradation process. a, The hydrophilic BC film and **b**, The superhydrophobic meta-skin in the enzyme solution.

It has been reported in previous literatures that the cellulase is capable of degrading cellulose into glucose owing to the decomposition of β-1, 4 glycosidic bonds[1]. Supplementary Fig. 4 presented the degradation process of hydrophilic BC film and superhydrophobic BC meta-skin. From the result, it is apparent that the hydrophilic BC film was completely degraded within 4 h. However, it spent nearly 16 h to degrade superhydrophobic BC meta-skin completely due to the nanoparticle decoration. Due to superhydrophobicity of the meta-skin, there exists an air layer between the meta-skin and HAc-NaAc buffer, avoiding the contact between cellulase and BC fibers. As a result,

only a spot of nano-fibers was degraded by cellulase at the initial stage. With escaping of SiO_2 nanoparticles, more nanofibers were exposed to the HAc-NaAc buffer, accelerating the degradation processes. In summary, the superhydrophobic BC meta-skin possesses degradability, although the nanoparticle decoration will decelerate the biodegradation process.

Supplementary Note 3. Theoretical and simulation of BC meta-skin

I. *Model of superhydrophobic surfaces*

Generally, the meta-skin surface is very rough due to superhydrophobic property. The surface roughness is conformed to the Gaussian distribution, which is expressed as[2]

$$R_f = \frac{1}{A_{wf}} \iint \sqrt{1 + \left(\frac{\partial z}{\partial x}\right)^2 + \left(\frac{\partial z}{\partial y}\right)^2}\, dxdy$$

$$= \sqrt{1 + 2\frac{\sigma^2}{L^2} \times \frac{[\exp(-\frac{\beta}{L})]^2}{\pi}}, \qquad \text{(S1)}$$

where R_f is the surface roughness, β is the correlation length, L is the characterization step length of the surface morphology, and σ is the standard deviation.

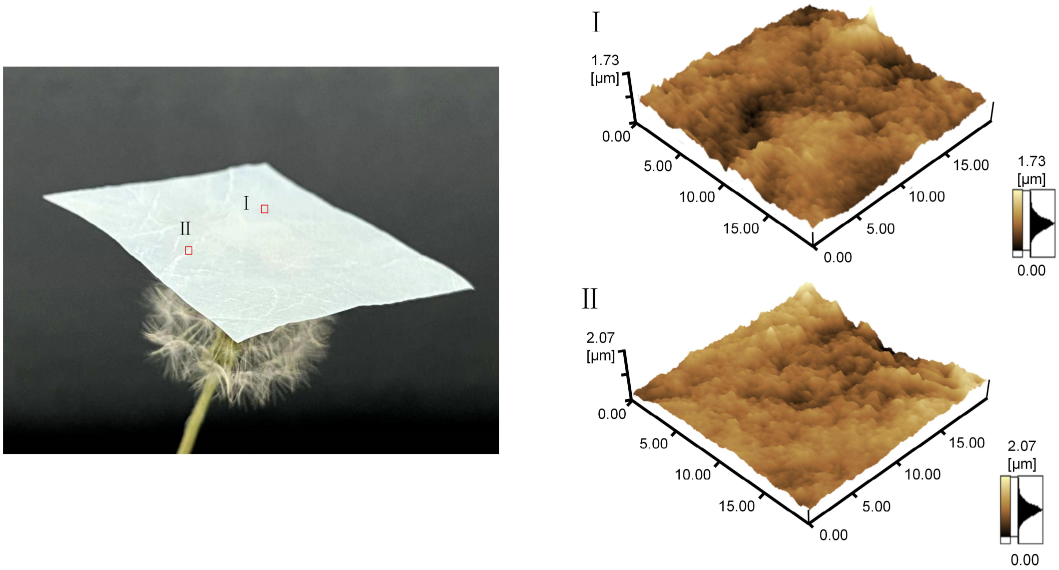

Supplementary Fig. 5. Surface roughness of BC meta-skin. We randomly chose two positions (I and II) on the meta-skin to characterize the surface roughness by using the scanning probe microscope.

From the measurement results of the scanning probe microscope (SPM-9700) in Supplementary Fig. 5, we can extract the parameters in Eq. (S1). In this example, the measurement step length L=0.2 μm, the average surface roughness R_f=1.9 μm, and the standard deviation σ=0.44 μm. Using these parameters, the correlation length β is calculated to be almost zero, indicating that the meta-skin surface is rather rough, which is one of the necessary conditions for superhydrophobicity.

II. *Simulation of total ultrasound reflection*

Due to the superhydrophobic nature of the meta-skin, stable Cassie-Baxter states (*viz*., the micro-sized air bubbles) form on the rough nanosurface. We assume that the thickness of air layer in water is equal to the surface roughness of meta-skin. Therefore, in numerical simulations, we set the size of air microbubbles to be 1.9×1.9 μm^2 and the period of bubble array is 3.8 μm for a simplified model. The background is water. As shown in Supplementary Fig. 6(a), total ultrasound reflection occurs at the air/water interface due to a large impedance mismatching in the frequency range from 0.3 to 5.4 MHz, with the transmission less than −19.4 dB, in agreement with the experimental measurement. In Supplementary Fig. 6(b), the calculated intensity field distributions show that the transmittance of ultrasound through the meta-skin decreases with increasing frequencies.

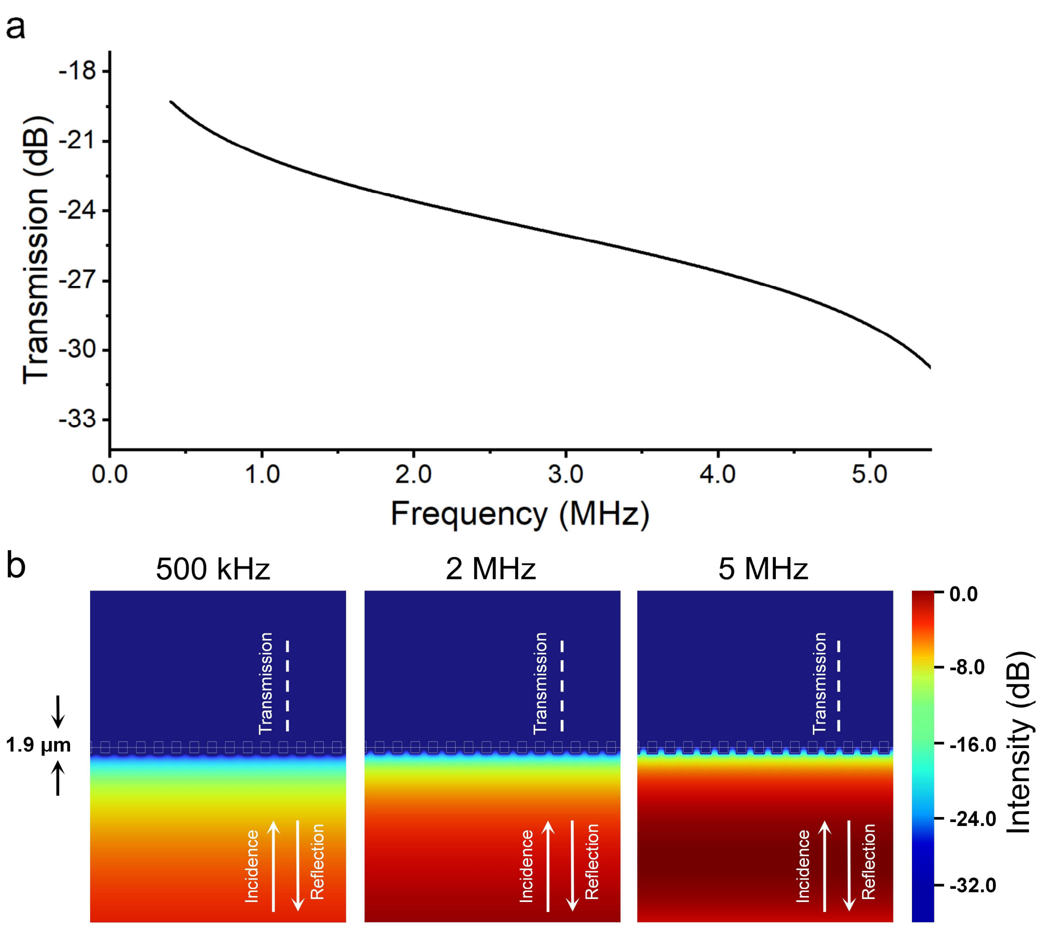

Supplementary Fig. 6. The reflection/transmission simulation. a, Transmission of ultrasound impinging on the BC meta-skin in the frequency range of 0.3~5.4 MHz. **b**, Intensity distributions of ultrasound at 0.2 MHz, 2 MHz, and 5.0 MHz. In the numerical simulation, the size of air microbubble is 1.9×1.9 μm^2 and the period of bubble array is 3.8 μm. The ultrasound beams are incident from below and the transmission through the microbubble array is close to zero.

Supplementary Note 4. Theory and experiment of acoustic holography

I. *Theory*

A single-scattering process can be described by the Fredholm integral equation, for example, the Lippmann-Schwinger equation in the wave scattering problem

$$\phi_{\boldsymbol{k}}^{\mathrm{diff}} = \exp(i\boldsymbol{k}\cdot\boldsymbol{r}) + \int d^3\boldsymbol{r}_1 G_+(\boldsymbol{r}-\boldsymbol{r}_1)u(\boldsymbol{r}_1)\phi_{\boldsymbol{k}}^{\mathrm{diff}}, \quad (S2)$$

where $\phi_{\boldsymbol{k}}^{\mathrm{diff}}$ represents the diffraction mode, with $\boldsymbol{k}$ the wave vector of incident wave

and $\exp(i\boldsymbol{k}\cdot\boldsymbol{r})$ the incident wave. $G_+(\boldsymbol{r}-\boldsymbol{r}_1)$ is the outward Green's function of the Helmholtz equation, which satisfies

$$(\nabla^2+\boldsymbol{k}^2)G_+(\boldsymbol{r}-\boldsymbol{r}_1)=\delta(\boldsymbol{r}-\boldsymbol{r}_1). \tag{S3}$$

In Eq. (S2), $u(\boldsymbol{r}_1)$ is the redefined potential energy of the scatterer, which possesses the $[\mathrm{m}^{-2}]$ dimension. If we consider the first-order approximation of Lippmann-Schwinger equation

$$\phi_{\boldsymbol{k}}^{\mathrm{diff}}=\exp(i\boldsymbol{k}\cdot\boldsymbol{r})+\int d^3\boldsymbol{r}_1 G_+(\boldsymbol{r}-\boldsymbol{r}_1)u(\boldsymbol{r}_1)\exp(i\boldsymbol{k}\cdot\boldsymbol{r}_1), \tag{S4}$$

the equation indicates that wave scattering state is a superposition of incident excitation wave and the wavelets from the scatterer excited by incident wave, which is also termed the 'Huygens principle'. Then if we further consider the second-order approximation of the Lippmann-Schwinger equation

$$\phi_{\boldsymbol{k}}^{\mathrm{diff}}=\exp(i\boldsymbol{k}\cdot\boldsymbol{r})+\int d^3\boldsymbol{r}_1 G_+(\boldsymbol{r}-\boldsymbol{r}_1)u(\boldsymbol{r}_1)\exp(i\boldsymbol{k}\cdot\boldsymbol{r}_1)$$
$$+\int d^3\boldsymbol{r}_1 d^3\boldsymbol{r}_2 G_+(\boldsymbol{r}-\boldsymbol{r}_1)u(\boldsymbol{r}_1)G_+(\boldsymbol{r}_2-\boldsymbol{r}_1)u(\boldsymbol{r}_2)\exp(i\boldsymbol{k}\cdot\boldsymbol{r}_2), \tag{S5}$$

we will obtain the wave scattering state that considers the secondary wavelets from the wavelets excited by the incident wave. Analogously, taking the integral progression into consideration, the original Lippmann-Schwinger equation can be retrieved. It is obvious that the wave function scattering problem in quantum mechanics has a similar physical picture with the acoustic wave scattering problem. Therefore, such an integral equation can be used to deal with the problems which involve the acoustic wave scattering on various cases, such as through a perforated 2D surface.

Here we consider the case of 2D scatterers, which is a perforated 2D surface inside a homogeneous background medium. The potential energy of a 2D scatterer is difficult to be defined. However, according to the Huygens principle and the above discussions, the integrand term can be replaced by the point-source wave function, due to the similar response characteristics. Therefore, the integral equation of acoustic pressure field can be derived by

$$p(\boldsymbol{r})=p_{\mathrm{i}}{}'(\boldsymbol{r})+\frac{1}{V}\int d^3\boldsymbol{r}_1 ikG(\boldsymbol{r}-\boldsymbol{r}_1)S_0 p(\boldsymbol{r}_1), \tag{S6}$$

where $p(\boldsymbol{r})$ denotes the acoustic pressure that satisfies the asymptotic condition when

the 2D scatterers exist. In Eq. (S6), $p_{\mathrm{i}}(\boldsymbol{r})$ represents the incident wave pressure, while $ikG(\boldsymbol{r}-\boldsymbol{r}_1)S_0 p(\boldsymbol{r}_1)$ denotes the wavelet excited by incident waves. V is the volume of scatterers. S_0 is the superficial area of a point source. Because of the boundedness of scatterers and the propagation characteristics of point sources, the acoustic pressure in the whole space can be obtained based on its distribution on the scatterers. When we only consider the scattered acoustic wave, the incident wave term $p_{\mathrm{i}}'(\boldsymbol{r})$ can be taken off. For Eq. (S6), the integral equation can be solved through discretizing and with the help of linear algebra. Here, we discretize the surface and divide it into identical unit squares to obtain a discretization equation from Eq. (S6)

$$p^{(I)} = \sum_J [\delta_{IJ} + ik\xi^{(I)}(1-\delta_{IJ})G(\boldsymbol{r}^{(I)};\boldsymbol{r}^{(J)})S_0]p^{(J)}, \quad \text{(S7)}$$

$$G(\boldsymbol{r}^{(I)};\boldsymbol{r}^{(J)}) = \frac{e^{j\boldsymbol{k}\cdot(\boldsymbol{r}^{(J)}-\boldsymbol{r}^{(I)})}}{4\pi|\boldsymbol{r}^{(J)}-\boldsymbol{r}^{(I)}|}. \quad \text{(S8)}$$

where $p^{(I)}$ denotes the acoustic pressure on the unit I^{th}, S_0 is the area of each unit, $G(\boldsymbol{r}^{(I)};\boldsymbol{r}^{(J)})$ is the Green's function between two units $\boldsymbol{r}^{(I)}$ and $\boldsymbol{r}^{(J)}$, $p^{(J)}$ is the acoustic pressure from the unit J^{th}, and δ_{IJ} is the Kronecker symbol that indicates the self-radiation of unit I^{th} when $I=J$. In Eq. (S7), $\xi^{(I)}$ represents the characteristics of unit I^{th}. Specifically, when unit I^{th} is a through-hole, $\xi^{(I)}=1$; when unit I^{th} is a hard or soft boundary, we have $\xi^{(I)}=0$. Then, the reflected acoustic field and the transmitted acoustic field can be considered separately by

$$\begin{cases} p_{\mathrm{r}}^{(I)} = \sum\limits_J [\delta_{IJ} + ik\xi^{(I)}(1-\delta_{IJ})G(\boldsymbol{r}^{(I)};\boldsymbol{r}^{(J)})S_0]p_{\mathrm{r}}^{(J)}, \\ p_{\mathrm{t}}^{(I)} = \sum\limits_J [\delta_{IJ} + ik\xi^{(I)}(1-\delta_{IJ})G(\boldsymbol{r}^{(I)};\boldsymbol{r}^{(J)})S_0]p_{\mathrm{t}}^{(J)}. \end{cases} \quad \text{(S9)}$$

The reflected and transmitted acoustic pressure fields have a relation with the incident pressure field

$$\boldsymbol{p}_{\mathrm{i}} = \boldsymbol{p}_{\mathrm{r}} + \boldsymbol{p}_{\mathrm{t}}, \quad \text{(S10)}$$

where acoustic pressure vectors are expressed by $\boldsymbol{p}_{\mathrm{i}} = \left(p_{\mathrm{i}}^{(1)}, p_{\mathrm{i}}^{(2)}, \dots, p_{\mathrm{i}}^{(N)}\right)^{\mathrm{T}}$, $\boldsymbol{p}_{\mathrm{r}} = \left(p_{\mathrm{r}}^{(1)}, p_{\mathrm{r}}^{(2)}, \dots, p_{\mathrm{r}}^{(N)}\right)^{\mathrm{T}}$, and $\boldsymbol{p}_{\mathrm{t}} = \left(p_{\mathrm{t}}^{(1)}, p_{\mathrm{t}}^{(2)}, \dots, p_{\mathrm{t}}^{(N)}\right)^{\mathrm{T}}$. Substituting Eq. (S8) into Eq.

(S9), we can obtain

$$\boldsymbol{p}_{\mathrm{i}} = \boldsymbol{S}\boldsymbol{p}_{\mathrm{r}} + \boldsymbol{S}\boldsymbol{p}_{\mathrm{t}}, \tag{S11}$$

$$S^{(IJ)} = \left[\delta_{IJ} + ik\xi^{(I)}\left(1 - \delta_{IJ}\right)G\left(\boldsymbol{r}^{(I)}; \boldsymbol{r}^{(J)}\right)S_0\right]. \tag{S12}$$

In Eq. (S11), $\boldsymbol{S}$ is the scattering matrix originating from Eq. (S7). Considering the continuity of acoustic particle velocity field, we will have

$$\boldsymbol{u}_{\mathrm{i}} = \boldsymbol{u}_{\mathrm{r}} + \boldsymbol{u}_{\mathrm{t}}, \tag{S13}$$

which can be rewritten with acoustic pressure

$$\frac{\boldsymbol{p}_{\mathrm{i}}}{\rho c} = -\frac{\boldsymbol{p}_{\mathrm{r}}}{\rho c} + \frac{\boldsymbol{p}_{\mathrm{t}}}{\rho c}, \tag{S14}$$

where ρ is the mass density of the background medium and c is the velocity of sound in the background medium. Then the final expression of transmitted acoustic pressure and reflected acoustic pressure can be obtained from Eq. (S11) and Eq. (S13)

$$\begin{cases} \boldsymbol{p}_{\mathrm{t}} = \dfrac{1}{2}\left(\boldsymbol{E} + \boldsymbol{S}^{-1}\right)\boldsymbol{p}_{\mathrm{i}}, \\ \boldsymbol{p}_{\mathrm{r}} = \dfrac{1}{2}\left(-\boldsymbol{E} + \boldsymbol{S}^{-1}\right)\boldsymbol{p}_{\mathrm{i}}. \end{cases} \tag{S15}$$

In Eq. (S14), $\boldsymbol{E}$ denotes the identity matrix. Therefore, the acoustic field in the whole space due to the 2D scattering can be calculated. Also based on the genetic algorithms, we can retrieve the best configuration of 2D scatterers (*e.g.*, the through-hole array) for a preset pressure field distribution, thus realizing the meta-skin holography based on meta-skin paper-cutting.

II. *Experiment*

We have measured the incident and reflected pulses of ultrasound on the meta-skin at the central frequency of 500 kHz (or 0.5 MHz). The acoustic setup is shown in Supplementary Fig. 7(a). The ultrasound pulse was launched by the bottom transducer. For measuring the incident pulse, the ultrasound signal was received by the top transducer directly without setting the BC meta-skin in the propagation path. For measuring the reflected and transmitted pulses, ultrasound signals were received by the bottom and top transducers respectively by setting the BC meta-skin in the propagation path, where the received signals were actually reflected from or transmitted through the

meta-skin surface. In Supplementary Fig. 7(b), we performed the time-domain measurements for both incident and reflected pulses, where the reflected pulse was compensated by π phase for comparison with the incident one. In Figs. S7(c) and 7(d), the spectral analyses show that incident ultrasound pulse has been completely reflected in broadband and the reflectivity reaches over 97%.

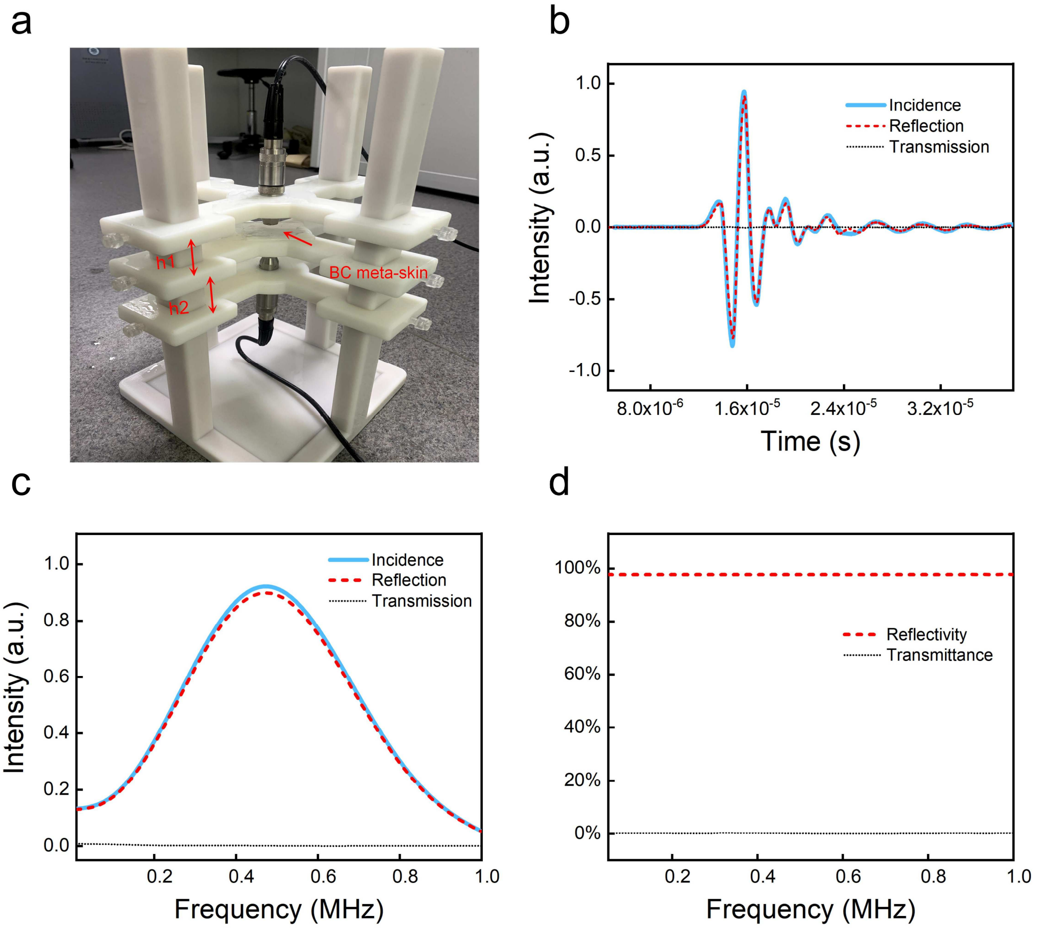

Supplementary Fig. 7. Reflection and transmission of BC meta-skin at 0.5 MHz. a, Acoustic setup for measuring the incident, reflected and transmitted ultrasound pulses from BC meta-skin. The ultrasound pulse is launched by the bottom transducer. We set h_1=h_2 to make sure that incident, reflected and transmitted pulses are in the same time slot. **b**, The time-domain measurement of incident, reflected, and transmitted pulses, where the reflected pulse is compensated by π phase due to the soft-boundary-like surface of BC meta-skin. **c**, Spectral analyses of the incident, reflected, and transmitted signals. **d**, Reflectivity and Transmittance of ultrasound through BC meta-skin.

As shown in Supplementary Fig. 8, the acoustic holographic experiments were conducted by using an ultrasound field scanning system (P. A. UMS3). The piezo-transducer (Olympus) has a center frequency of 0.5 MHz. The ultrasound signal used in our experiment was a 5-cycle sine wave function. The ultrasound field emitted by the transducer was modulated by the holographic meta-lens and then scanned by a pin hydrophone (NH0500 Diameter = 500 μm).

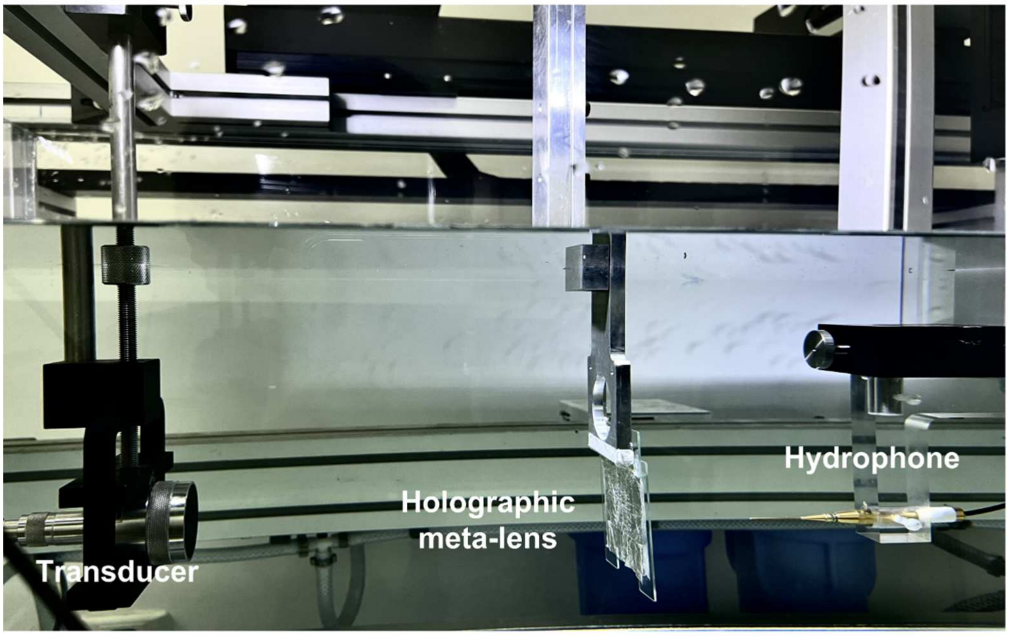

Supplementary Fig. 8. Acoustic set-ups for the holography experiment. The size of the holographic meta-lens is 30×30 mm^2. The spacing between the meta-lens and the hologram plane is 35 mm.

Supplementary Note 5. Design principle of 3D ultrasound imaging

I. *Theory*

According to the Huygens-Fresnel principle[3, 4], diffracted waves can be regarded as a superposition of propagating secondary wavelets in free space. For the sound wave, the exact expression of pressure field at any point in real space can be given by using the Helmholtz-Kirchhoff integral theorem. By setting the boundary conditions, we can derive the diffracted sound pressure fields from the wave equation and Green's theorem.

However, the Kirchhoff's diffraction theory violates the uniqueness theorem. Therefore, for the case of a flat diffractive screen (or the source plane), we employ the Rayleigh-Sommerfield diffraction integral[5].

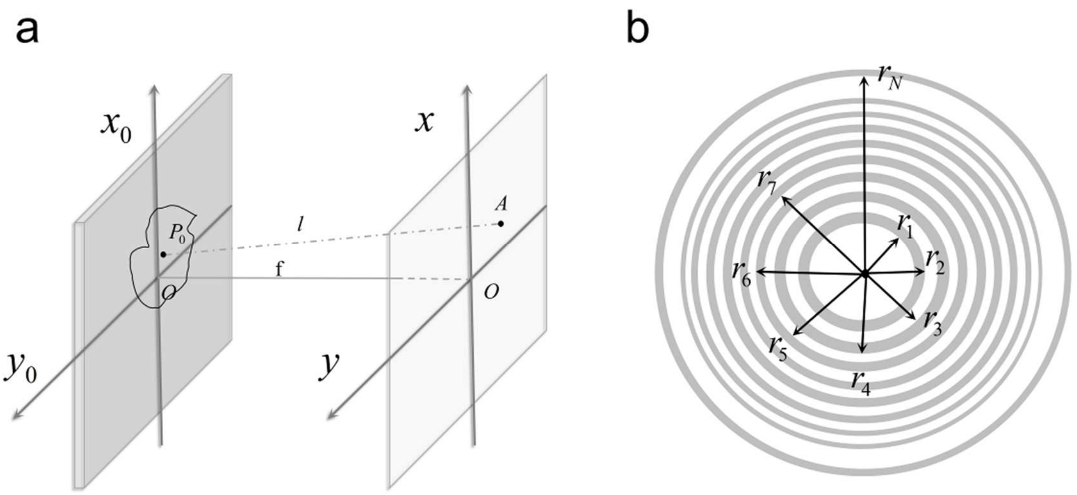

Supplementary Fig. 9. Diffraction theory and the design of an imaging meta-lens. **a**, Illustration of the Rayleigh-Sommerfeld diffraction. **b**, A schematic of the Fresnel zone plate design.

As shown in Supplementary Fig. 9, the x_0y_0 plane is where the sound source is located. Here P_0 is an arbitrary point (x_0, y_0) on the source plane. Then the sound pressure at the point $A(x, y)$ in the hologram plane can be expressed as

$$P(A) = \frac{1}{i\lambda}\iint P(P_0)\,K(\theta)\,\frac{\exp(ikl)}{l}\,dS. \qquad \text{(S16)}$$

The response function of the system is

$$h(A, P_0) = \frac{1}{i\lambda}K(\theta)\frac{\exp(ikl)}{l}. \qquad \text{(S17)}$$

Equation (S16) can be further expressed into

$$P(x, y) = \frac{1}{i\lambda}\iint P(x_0, y_0)\,h(x, y; x_0, y_0)dx_0dy_0, \qquad \text{(S18)}$$

indicating a linear system with $K(\theta) \approx 1$ under the paraxial approximation condition. Therefore, the impulse response function of the linear system can be simplified into

$$h(x, y; x_0, y_0) = \frac{1}{i\lambda}\frac{\exp(ikl)}{l} = \frac{1}{i\lambda}\frac{\exp\left[\left(ik\sqrt{f^2 + (x - x_0)^2 + (y - y_0)^2}\right)\right]}{\sqrt{f^2 + (x - x_0)^2 + (y - y_0)^2}}$$

$$= h(x - x_0, y - y_0)\,. \qquad \text{(S19)}$$

Equation (S19) also shows that the impulse response function has a spatially invariant form in a linear invariant system, which provides a basis for analyzing the diffraction phenomenon as well as the design of the Fresnel zone plate.

II. *Experiment*

The focusing behavior of the Fresnel zone plate is based on the principle of wave diffraction. When the incident acoustic wave is modulated by the zone plate, the phase difference of wavelets reaching the focal point is an integer multiple of 2π, resulting in the constructive superposition. As shown in Supplementary Fig. 9(a), by setting the focal point f, the geometric parameters of the zone plate r_n can be expressed as

$$r_n = [(f + \frac{n\lambda}{2})^2 - f^2]^{\frac{1}{2}}, \tag{S20}$$

where λ is the wavelength of ultrasound. From Eq. (S20), as the value of n increases, the strip width of meta-lens becomes smaller, even reaching 175 μm for the narrowest strip. This requires good machinability of BC meta-skin and the accuracy of paper-cutting. The fabricated sample is shown in Supplementary Fig. 10(b), which indicates an ultralight weight, just like a piece of hollow-out paper, which is distinctive in the field of meta-surfaces.

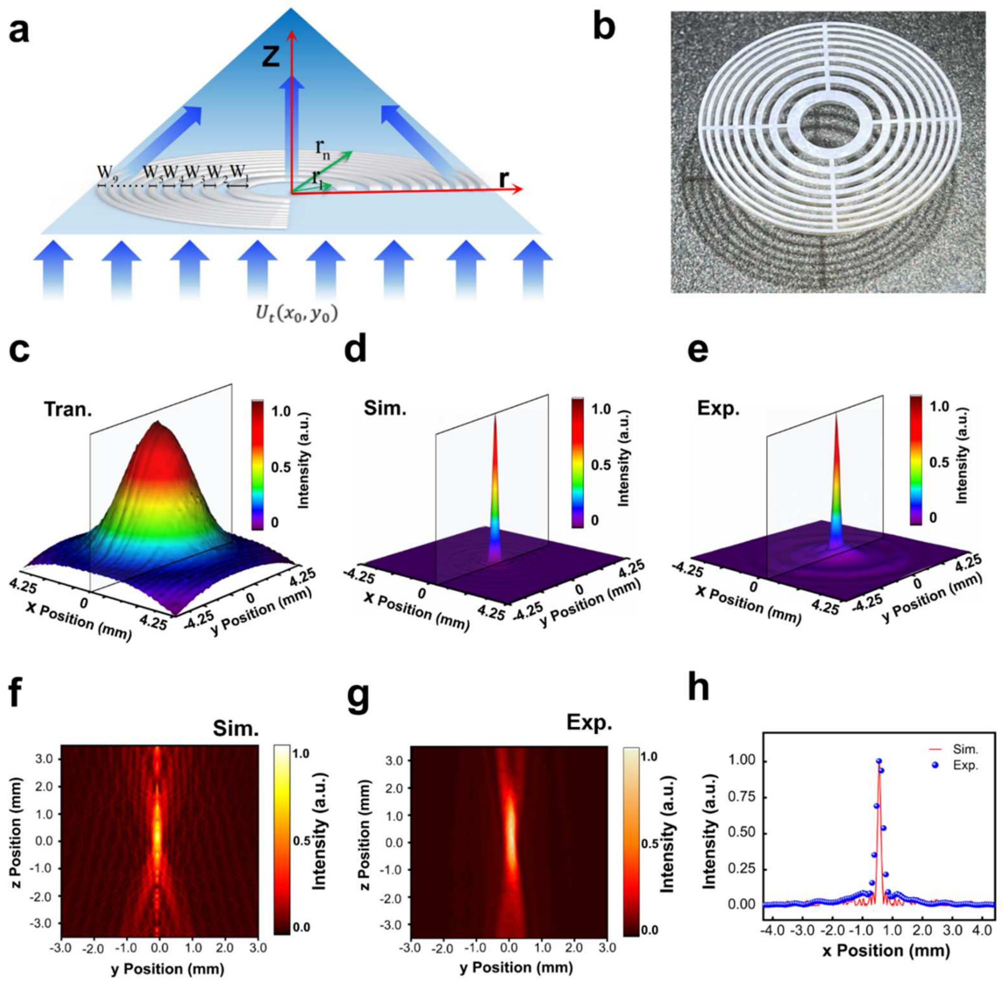

Supplementary Fig. 10. The meta-skin paper-cutting based ultrasonic flat lens. a, A schematic of the focusing Fresnel meta-lens. **b**, Photograph of the fabricated skin meta-lens. **c**, The ultrasound intensity field generated directly by the unfocused planar transducer in the experiment. **d**, Simulation of the intensity field in the *x*-*y* plane after the focusing of the skin meta-lens. **e**, Experimental result of the intensity field in the *x*-*y* plane after the focusing of the skin meta-lens. **f**, Simulation of the intensity field in the *y*-*z* plane after the focusing of the skin meta-lens. **g**, Experimental result of the intensity field in the *y*-*z* plane after the focusing of the skin meta-lens. **h**, The intensity profiles along the line *x*=0 in **d** and **e**, where the simulation and experimental results show that FWHM (Sim.) = 185 μm and FWHM (Exp.) = 195 μm.

The operation frequency of planar transducer used in the experiment was 5 MHz. A 10-cycle sinusoidal ultrasound signal was launched and the intensity field at *z*=19

mm was measured. The experimentally scanned intensity field was shown in Supplementary Fig. 10(c) with FWHM=6.2 mm. To show the focusing property of meta-lens, we conducted the simulation by using COMSOL software and did the experiment by using an ultrasound field scanning system. The simulated intensity field distributions in *x*-*y* plane and *y*-*z* plane are presented in Supplementary Figs. 10(d) and 10(f). In the simulations, the FWHM of the focal spot is 185 μm and the focal length is 940 μm. The measured intensity field distributions in *x*-*y* plane and *y*-*z* plane are shown in Supplementary Figs. 10(e) and 10(g). In the experiment, the measured FWHM of the focal spot is 195 μm and the focal length is 1120 μm. Supplementary Figure 10(h) shows the quantitative comparison between the transverse sizes of focal spots in simulation and experiment, which agrees with each other. In conclusion, the BC meta-skin paper-cutting is a perfect candidate for implementing ultrathin and superlight ultrasonic imaging meta-lens.

Supplementary Note 6. Lateral and longitudinal resolutions in imaging

Resolution refers to the ability to distinguish two objects segregated in a distance. High resolution indicates that the imaging process can distinguish two objects with a small space between. In ultrasound imaging, two types of resolutions are commonly used, *viz*., lateral and longitudinal resolutions. Lateral resolution, known as azimuth or radial resolution, describes the resolution perpendicular to the propagating axis of the beam. Longitudinal resolution, also known as range or axial resolution, describes the resolution along the propagating axis of the beam.

The lateral resolution is mainly determined by the spot size of a focused ultrasound beam, as shown in Supplementary Fig. 11(a). Here we set the spot size of the focused beam to be d_1 and the distance between two objects A and B also to be d_1. When the focused beam is swept on the object A or B, there will be a strong echo signal received by the transducer. When the focused beam is swept on other areas, for example the space between objects A and B, the echo signal will be very weak. The images of objects A and B can thus be reconstructed through processing of echo signals. However, when

the distance between objects A and B is less than d_1, the focused beam cannot be used to distinguish the two targets, where the echo signals will identify two objects as one.

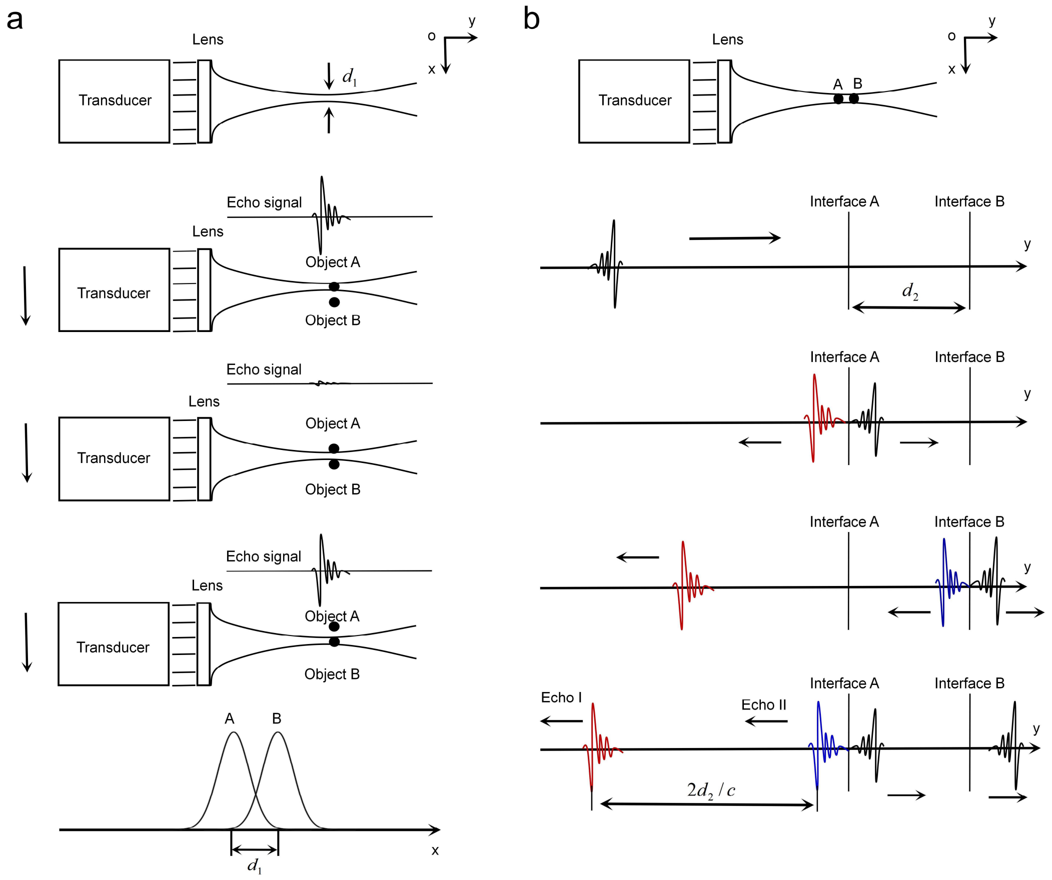

Supplementary Fig. 11. Schematics of the lateral and longitudinal resolutions for ultrasound imaging based on meta-skins. a, The lateral resolution for distinguishing objects A and B. **b**, The longitudinal resolution for distinguishing interfaces A and B.

In the propagation direction, the smallest distance between two adjacent interfaces one can distinguish is termed the longitudinal resolution (ΔR_l). After the ultrasound is modulated by the meta-lens, it passes through the interfaces A and B in sequence and generates echo signals I (red) and II (blue), respectively, as shown in Supplementary Fig. 11(b). When the echoes I and II do not overlap, we can distinguish interfaces A and B, for which the following formula must be satisfied

$$\tau \leq \frac{2d_2}{c}, \quad \text{(S21)}$$

where τ is the pulse width, c is the sound velocity, and d_2 is the interfacial separation.

Thus the following formula can be obtained

$$d_{min} = \Delta R_l = \frac{\tau c}{2}, \quad \text{(S22)}$$

where d_{min} is the smallest distance between two targets that can be distinguished, *viz.*, the longitudinal resolution. When the distance between two interfaces is less than ΔR_l, the imaging system cannot distinguish the two interfaces and mistakenly identifies them as one single interface. The pulse width τ is the main factor influencing the longitudinal resolution.

Supplementary Note 7. Technical recipes for ultrasound imaging

I. *Theory*

During the scanning of meta-lens's focal spot in the *x* and *y* directions, the received echo signal is $s(t)$. Here the peaks of $s(t)$ at different times contain the location information of objects at different depths and the contrast of acoustic impedance between the object and background medium. However, $s(t)$ cannot be directly used for the object reconstruction. It is necessary to demodulate the signal $s(t)$ to obtain $\hat{s}(t)$ for image reconstruction, where the Hilbert transform[6, 7] is

$$\hat{s}(t) = H[s(t)] = \int_{-\infty}^{\infty} \frac{s(\tau)}{t-\tau} d\tau = s(t) * \frac{1}{\pi t}. \quad \text{(S23)}$$

In Eq. (S23), $H[\,]$ represents the Hilbert transform, τ is the arrival time of echo signal, and $*$ denotes the convolution operator. In the experiment, the echo signal was sampled, so that $s(t)$ in a single-point measurement takes a matrix form of

$$s(t) = [A_1(t_1) \quad \cdots \quad A_N(t_N)]. \quad \text{(S24)}$$

In Eq. (S24), t_n is the time position of each sampled echo signal, which corresponds to the depth of imaging object by $z_n = t_n c$, where c is the speed of sound in water. A_n is the amplitude of sampled echo signal at t_n. N is the number of sampling points. We can perform the Hilbert transform on the matrix signal $s(t)$ to obtain the matrix form of $\hat{s}(t)$, which can be expressed as

$$\hat{s}(t) = s(t) * \frac{1}{\pi t}, \quad \text{(S25)}$$

or

$$\hat{s}(t) = [B_1(t_1) \quad \cdots \quad B_N(t_N)], \tag{S26}$$

where B_n contains the information of objects at different depths. Then, after scanning the field in x and y directions, we can obtain the 3D volume data $I(x, y, t)$ of the imaging objects in space

$$I(x, y, t) = \begin{bmatrix} \hat{s}(x_1, y_1, t) & \cdots & \hat{s}(x_m, y_1, t) \\ \vdots & \ddots & \vdots \\ \hat{s}(x_1, y_m, t) & \cdots & \hat{s}(x_m, y_m, t) \end{bmatrix}, \tag{S27}$$

where each sampling point $\hat{s}(x_n, y_n, t)$ contains a time-domain signal sequence of a length N. The time domain signal corresponds to the depth z_n of the image ($z_n = t_n c$.). The information of each section in the 3D volume data can be expressed as

$$I(x, y, z_n) = \begin{bmatrix} \hat{s}(x_1, y_1, t_n) & \cdots & \hat{s}(x_m, y_1, t_n) \\ \vdots & \ddots & \vdots \\ \hat{s}(x_1, y_m, t_n) & \cdots & \hat{s}(x_m, y_m, t_n) \end{bmatrix}, \tag{S28}$$

which is shown in the experiment section. We can also present the projection image of 3D objects on a 2D plane via the superposition of time-domain signals in Supplementary Figs. 12(c) and 13(f). The 3D data reconstruction processes for the cases in Supplementary Figs. 12 and 13 are shown in Supplementary Movies 5 and 6.

II. *Experiment*

As shown in Supplementary Fig. 12(a), the objects for ultrasound imaging were graphite rods, for which the projection pattern from the View 1 was a 'six-pointed star'. The 6 graphite rods were divided into three groups with two rods in each group being parallel and oriented in different directions. The six graphite rods in three groups were distributed in three planes 1, 2, and 3, respectively. The three planes were within the range of the focal spot, as shown in Supplementary Fig. 12(b). In the measurement process, the 3D volume data of the object image was obtained by just scanning the focal spot in the *x*-*y* plane (*z* axis is the propagation direction) and processing the echo signals. The result for the projection image in the *x*-*y* plane is shown in Supplementary Fig. 12(c). The 3D data reconstruction process by considering the time-domain echo signals is shown in Supplementary Movie 5.

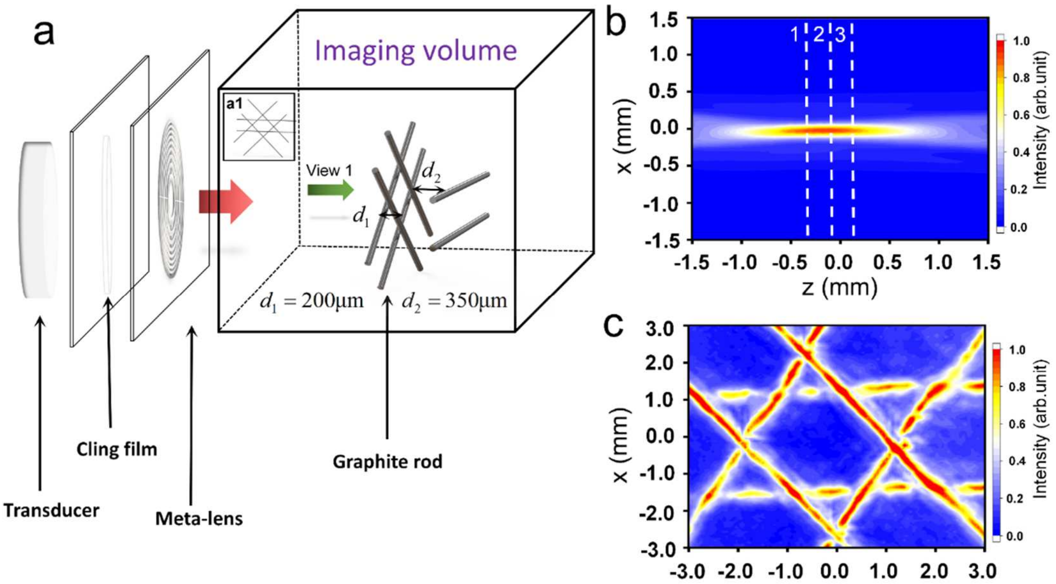

Supplementary Fig. 12. Meta-lens imaging for testing longitudinal resolution. a, Three groups of objects placed in sequence in the direction of sound propagation, where each group of objects is a pair of parallel graphite rods with the diameter of 200 μm and oriented in a certain direction. The inset shows the projection of graphite rods in View 1. **b**, The positions of three planes 1, 2, 3 in which the objects locate. The three positions are within the range of the focal spot. **c**, Imaging results without processing the time-domain echo signals, which only displays the measured projection of graphite rods in View 1.

The positions of imaging objects ‘HUST’ and ‘SIAT’ are shown in Supplementary Fig. 13(a). The imaging objects are metallic patterns of the thickness about 100 μm. In the experiment, the spacing between the object ‘HUST’ from the skin meta-lens was 7.4 mm, while the spacing between the object ‘SIAT’ from the meta-lens is 8.5 mm. The two planes where the two objects located were within the focal spot shown in Supplementary Fig. 13(b). The intensity fields along the dashed lines 1 and 2 in Supplementary Fig. 13(b) are shown in Supplementary Fig. 13(c). In addition, the intensity field distributions of foci in the planes of 1 and 2 correspond to the insets c1 and c2 of Supplementary Fig. 13(c), respectively. In the Supplementary Movie 6, we note that the image of “SIAT” appears from left to right, and the image of “HUST”

appears from the middle to two sides. In our experiment, the thickness of objects "HUST" and "SIAT" is only 100 μm, making the metallic objects flexible. During the assembling process, the objects "HUST" and "SIAT" are slightly distorted to a certain extent, as shown in Supplementary Fig. 13(a). For example, the object "SIAT" has a slight inclination to the right side. The object "HUST" is bent with the central part curved toward the transducer. This slight distortion with an amplitude of several hundred micrometers can be distinctively rendered in the echo ultrasound signals, which also demonstrates the high longitudinal resolution of our approach.

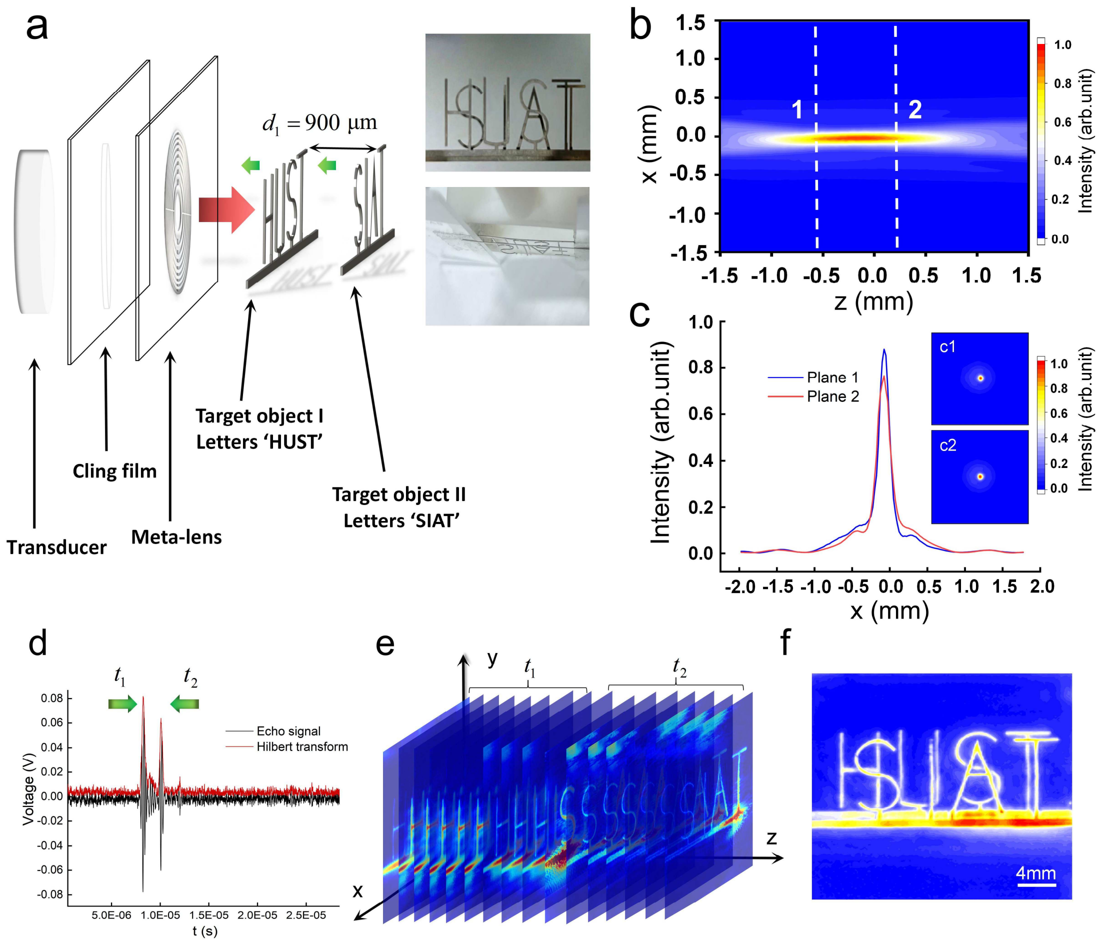

Supplementary Fig. 13. Meta-lens imaging for testing the transverse resolution. a, Illustration of 3D echo imaging with the meta-lens, where the imaging objects are the metallic patterns of 'HUST' and 'SIAT' as shown in the inset. **b**, Focused ultrasound intensity field along the propagating axis, where the objects 'HUST' and 'SIAT' locate in the planes 1 and 2, respectively. **c**, Intensity field distributions of foci in the planes of 1 and 2, corresponding to the insets of c1 and c2, respectively. **d**, The time-domain

echo signal acquired in a single measurement. **e**, 3D volume data constructed from the scanned field. **f**, Echo image after the superposition of time-domain signals.

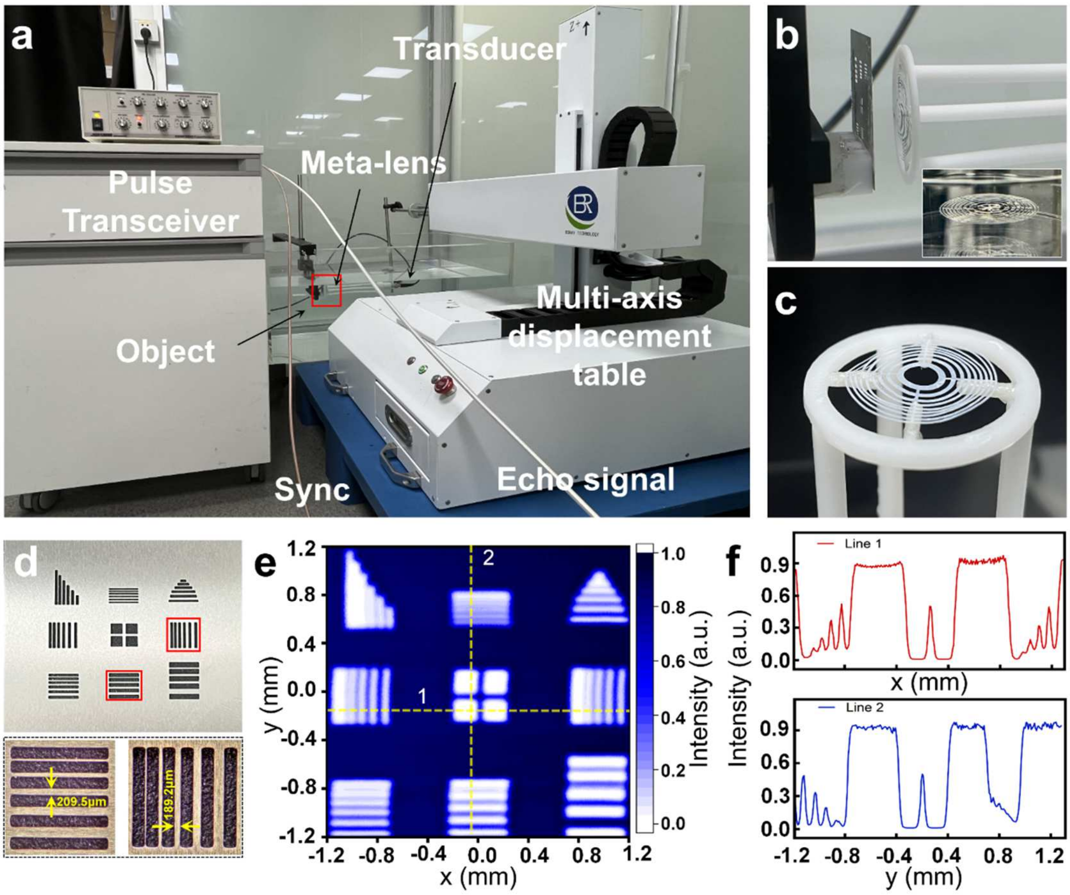

Supplementary Fig. 14. Acoustic setups for echo imaging. a, Acoustic setups used in experiments. **b**, Photograph of a meta-skin lens set for imaging. Inset shows the meta-lens immersed in water with a silvery surface. **c**, Zoom-in picture of the meta-lens. **d**, The lateral-resolution testing board, where the width of the narrowest slit is 50 μm. **e**, The ultrasonic imaging result of the testing board. **f**, The image intensity profiles along the lines 1 and 2 in **e**, respectively.

In the echo imaging experiment, a pulse transmitter/receiver (JSR-DPR-300) was used to transmit and receive the broadband pulses, which were then finely modulated and focused by the skin meta-lens for the echo imaging [Supplementary Fig. 14(a)]. When the meta-lens was set on the framework and immersed in water, the surface will become silvery due to the formation of stable air/fiber interface, as shown in

Supplementary Figs. 14 (b) and 14 (c). In Supplementary Fig. 14(d), we presented a standard resolution etalon for characterizing the echo imaging, for which the minimum spacing is 50 μm. The standard plate was scanned by moving the focal spot of imaging system, and the echo imaging result was shown in Supplementary Fig. 14(e). Due to the diffraction limit, the minimum interval that the imaging system can resolve is about 189.2 μm. We also show the intensity distributions along the lines 1 and 2 in Supplementary Fig. 14(e), for which the results are displayed in Supplementary Fig. 14(f) for better characterizing the resolution of imaging. Compared with meta-lenses made of gratings or gradient metamaterials, our proposed meta-skin paper-cutting lens is ultralight and ultrathin. In terms of performance, the lateral resolution can reach 0.63λ, which is close to the Rayleigh diffraction limit. More importantly, the meta-skin lens is flexible and compatible with hydrogels, which can image objects inside curved and rough surfaces.

Supplementary References

1. Chen, K. *et al*. $CoFe_2O_4$ Embedded Bacterial Cellulose for Flexible, Biodegradable, and Self-powered Electromagnetic Sensor. *Nano Energy.* **102,** 107740 (2022).
2. Tong, L. et al. An Acoustic Meta-Skin Insulator. *Adv. Mater.* **32**, 2002251 (2020).
3. Goussev, A. Huygens-Fresnel-Kirchhoff construction for quantum propagators with application to diffraction in space and time. *Phys. Rev. A* **85**, 013626 (2012).
4. Makris, K.G. & Psaltis, D. Huygens-Fresnel diffraction and evanescent waves. *Opt. Commun.* **284**, 1686-1689 (2011).
5. Charnotskii, M. Extended Huygens-Fresnel principle and optical waves propagation in turbulence: discussion. *JOSA A* **32**, 1357-1365 (2015).
6. Cizek, V. Discrete hilbert transform. *IEEE Trans. Audio Electroacoust.* **18**, 340-343 (1970).
7. Liu, Y.-W. Hilbert transform and applications. *Fourier Transform Applications*, 291-300 (2012).
