# Supplementary material for: Decorated bacteria-cellulose ultrasonic metasurface
Source: Nat Commun. 2023 Sep 1;14:5319. doi: 10.1038/s41467-023-41172-2 (PMC10474036; doi:10.1038/s41467-023-41172-2)
Supplement: Supplementary file 3 — Description of Additional Supplementary Files [file 41467_2023_41172_MOESM3_ESM.pdf]

Description of Additional Supplementary Files:

Supplementary Movie 1: The process of droplets falling on BC meta-skin captured by a high-speed camera.

Supplementary Movie 2: The ejection of BC meta-skin due to the ultra-low surface energy.

Supplementary Movie 3: The superhydrophobic property of the BC meta-skin after active self-healing.

Supplementary Movie 4: The ultralight BC meta-skin resting on the corolla of dandelion.

Supplementary Movie 5: 3D reconstruction of imaging objects 1 (longitudinal resolution).

Supplementary Movie 6: 3D reconstruction of imaging objects 2 (transverse resolution).
