# Supplementary material for: Decorated bacteria-cellulose ultrasonic metasurface
Source: Nat Commun. 2023 Sep 1;14:5319. doi: 10.1038/s41467-023-41172-2 (PMC10474036; doi:10.1038/s41467-023-41172-2)
Supplement: Supplementary file 11 — Source code [file 41467_2023_41172_MOESM11_ESM.pdf]

```
function [ fitness ] =
fitnessfunc_udwt( v )
%pixel_a=3.75E-4;              %%m
pixel_n=8E1;
nu=5E5;                   %%Hz
c0=1.5E3;                   %%m/s
lambda=c0/nu;
k=2*pi/lambda;
rho0=1E3;                  %%kg/m^3
S0=2*pixel_a^2;                %%m^2
%d_s=1E-1;                   %%m
d_i=3E-2;                   %%m

%v(:,1)=double(v);


%%
%%
m_v=reshape(v,pixel_n/2,pixel_n/2);
r_v=[m_v,fliplr(m_v);flipud(m_v),rot90(
m_v,2)];
v=r_v(:);%%
```

```
%%
%m_1=reshape(v,pixel_n/2,(pixel_n-30)/2)
;
%e1=eye((pixel_n-30)/2,(pixel_n-30)/2);
%e2=zeros((pixel_n-30),30/2);
%e=[e1,e2];
%m_2=m_1*e;
%ce=blkdiag(zeros((pixel_n-30)/2,(pixel
_n-30)/2),eye(30/2,30/2));
%m_3=ce*m_2;
%v_f=m+m_3'+blkdiag(zeros((pixel_n-30)/
2,(pixel_n-30)/2),ones(30/2,30/2));
%m_v=reshape(v_f,pixel_n/2,pixel_n/2);
%r_v=[m_v,fliplr(m_v);flipud(m_v),rot90
(m_v,2)];
%v=r_v(:);

%%
%
R=b_coordinate(pixel_a,pixel_n);
%%
```

```
%%Zxr
R_p=exp(-1i*k*R);
R_f=R-eye(pixel_n^2,pixel_n^2)*(-1i*k*r
ho0*c0*S0)/(2*pi);  %
func_G=R_p./(2*pi*R_f);
Zxr=1i*k*rho0*c0*func_G*S0;
M_th=diag(v);
Zxr=M_th*Zxr;
Zxr=Zxr-diag(diag(Zxr));
Zxr=rho0*c0*M_th+Zxr;
Zxr=Zxr-eye(pixel_n^2,pixel_n^2)+M_th;
clear R_p R_f ;
%%
%
u0=1E0;    %%m/s
%%
%R_s=s_coordinate( d_s,pixel_a,pixel_n );
    %%%%%%%%%%%
%func_G=exp(-1i*k*R_s)./(2*pi*R_s);
%Zs=1i*func_G*S0*k;
```

```
%u_in=Zs*u0*d_s./R_s;
%%
u_in=ones(pixel_n^2,1)*u0;              %
%%%%%%%%%%%%%%
%%
%
R_resis=diag(v);
intm=eye(pixel_n^2,pixel_n^2)-R_resis;
R_resis=R_resis*rho0*c0;
R_resis=R_resis+intm;
%%
%
I=eye(pixel_n^2,pixel_n^2);
u_tran=(1/2)*(I+Zxr\R_resis)*u_in;
R_r=sqrt(R.^2+d_i^2);
func_G=exp(-1i*k*R_r)./(2*pi*R_r);
Zst=1i*k*rho0*c0*func_G*S0;
p_tran=Zst*u_tran;
pr=abs(p_tran);
%i_p=reshape(pr,pixel_n,pixel_n);
```

```
%surf(i_p);view([0 0
1]);colormap(jet);shading interp;axis
off;
clear R fun_G;
%%
%
pr=reshape(pr,pixel_n,pixel_n);
image=mat2gray(real(pr));
%image=image(pixel_n/5+1:pixel_n-pixel_
n/5,pixel_n/5+1:pixel_n-pixel_n/5);
%%
%
image=255*image;
%rst=imbinarize(rst,100/255);
rst=double(image(:));
%%
%
A=imread('**\targetp.png');
Gray=rgb2gray(A);
%Gray=imbinarize(Gray,100/255);
```

```
goal=double(Gray(:));
fitness=((rst-goal).^2)';
fitness=sum(fitness(:));
fitness=sqrt(fitness);
fitness=gather(fitness);
end
```
